# Appearances and Evolution of a Recurrent Nora's Lesion of the Hand

**Published:** 2019-01-24

**Authors:** Ingrid Salna, Nicholas Solanki, Timothy Proudman

**Keywords:** Nora's lesion, bizarre parosteal osteochondromatous proliferation (BPOP), hand tumour, myositis ossificans

## CASE DESCRIPTION

**Figure 1:**
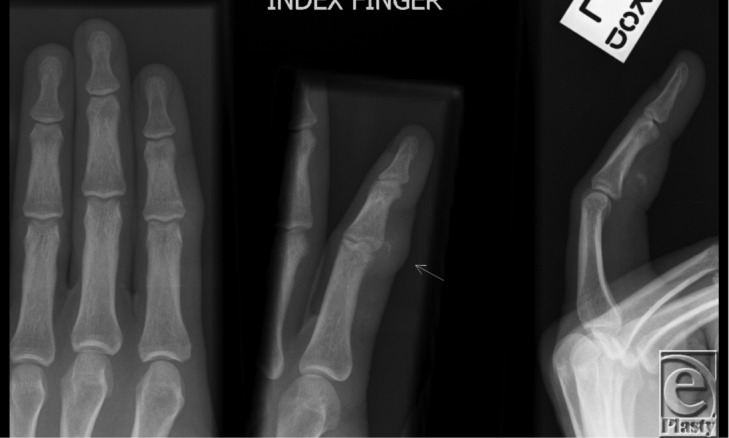
Initial radiograph showing soft-tissue prominence with a mostly radiolucent lesion with peripheral calcification volar to the base of the middle phalanx.

The case is of a 35-year-old right-hand-dominant man who worked in an office. He was a nonsmoker and had no significant medical comorbidities. He was referred with a lump on his left index finger several months following a simple laceration to the area from broken glass. The lump had been progressively enlarging, associated with tenderness and reduced finger flexion secondary to its size. The radiograph (Fig 1) showed soft-tissue prominence with a mostly radiolucent lesion with peripheral calcification volar to the base of the middle phalanx. On ultrasound scan, a relatively well-circumscribed mass was demonstrated with peripheral calcification just lateral to the flexor tendons measuring 14 × 7 × 13 mm. He subsequently underwent exploration in theater, where an encapsulated calcium-filled mass adherent to the middle phalanx and the A3 pulley was encountered. Histology revealed a benign cartilaginous proliferation in keeping with a soft-tissue chondroma. A diagnosis of Nora's lesion was considered but dismissed as the lesion lacked the typical zonal pattern consisting of central or basal new bone and a peripheral cap of cartilage.

**Figure 2:**
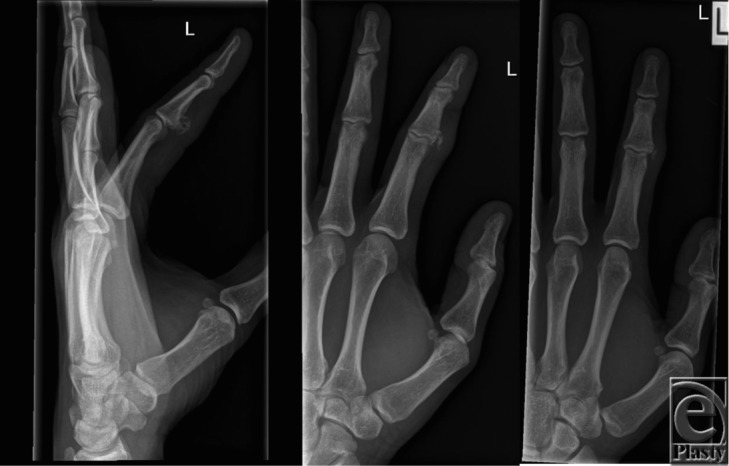
Radiograph at 6 months postoperatively, following initial exploration and removal. This shows a calcified mass at the previous operative site, separate from the underlying cortex.

At follow-up 6 months later, a recurrent mass was noted. The radiograph (Fig 2) showed recurrence of a calcified mass that appeared separate from the underlying cortex. He subsequently underwent reexploration and removal of a suspected recurrent enchondroma. Histology on this occasion was again consistent with a soft-tissue chondroma.

**Figure 3:**
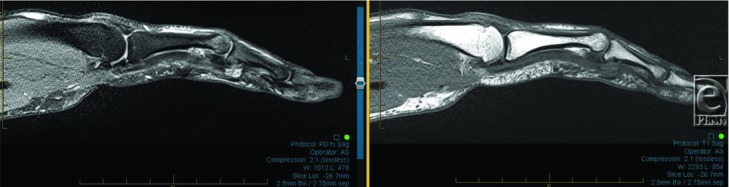
Lateral magnetic resonance scans at the second recurrence following excision showing the lesion on T1- and T2-weighted images.

**Figure 4:**
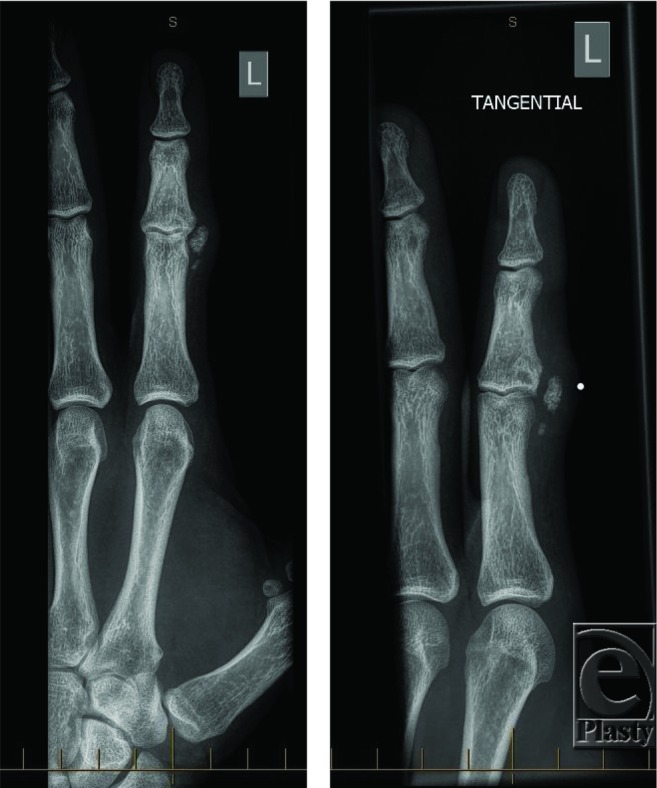
Radiograph at the second recurrence showing a calcified lesion along the volar and radial border of the proximal interphalangeal joint, the larger focus distally measuring up to 8 mm.

**Figure 5:**
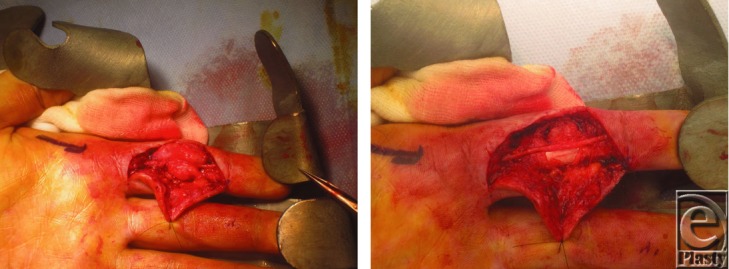
Intraoperative photographs taken after the second recurrence and third exploration, more than 2 years following the initial laceration.

He then presented with a second recurrence approximately 1 year later. On this occasion, magnetic resonance imaging (MRI) was performed in an attempt to delineate the lesion. The MRI revealed a nonspecific appearance of the lesion (Fig 3) but favored it to be a periosteal chondroma. Clinically, there was a mildly tender lump at the previous operative site with restriction in proximal interphalangeal joint (PIPJ) flexion. Repeat radiography (Fig 4) showed a more well-defined calcified lesion along the volar and radial border of the PIPJ, the larger focus distally measuring up to 8 mm. He underwent reexploration and marginal excision of the bony lesion. Intraoperative findings showed a bony mass arising from the proximal radial-volar base of the middle phalanx (Fig 5). Histology at this time reported the lesion to be Nora's lesion progressing to acquired exostosis. No clinical recurrence has been seen in the subsequent 6 months.

## QUESTIONS

1. What is Nora's lesion (or bizarre parosteal osteochondromatous proliferation)?
2. How is it diagnosed?
3. What is the cause?
4. How is it treated?

First described by Nora et al^1^ in 1983, it is a rare, benign, reactive mineralizing mesenchymal lesion with less than 200 cases reported in the literature. It usually affects small bones of the hand and feet,^1^^-^5 and epidemiologically it is more common in young patients in the second to fourth decades of life and affects both sexes equally.^1^^,^^2^^,^^4^^,^^6^ Clinically, there is usually a small mass separate from the overlying skin^1^ and the growth period may be variable, from months to years. In a few cases, the growth may be quite aggressive, and in a few cases, the lesion is even slightly painful mostly due to the mass effect and decreased range of motion, thus suggesting the clinical behavior of a malignant neoplasm.^7^^,^^8^ There is sometimes a history of trauma but not always.^8^

Nora's lesion can arise from bone or within soft tissue, and diagnosis is made by a combination of radiology and, most importantly, histological findings. Diagnosis can be difficult due to the varied appearances of the lesion at different stages of the disease. In addition, it can be easily confused with other lesions as there is some overlap in appearances with osteochondroma, periosteal chondroma, parosteal osteosarcoma, and chondrosarcoma and its high recurrence rate may lead to a mistaken diagnosis of malignancy. In this case, consecutive radiographs were available, showing the natural evolution of the lesion in keeping with other case reports.^6^ Radiologically, it appears as a well-demarcated, pedunculated or sessile tumor arising from the cortical surface of the underlying bone without disturbing its native architecture.^2^^,^^4^ In terms of the natural evolution, the first stage is parosteal soft-tissue swelling or mass, sometimes with tiny calcification. The second stage is seen as parosteal, flame-like calcifications, and the third stage is a matured osteophytic bony lesion, well-demarcated, pedunculated/sessile arising from cortical surface of the underlying bone without disruption of the native architecture.^6^ Recurrences have been described as partially calcified or completely ossified lesions with less homogeneous, more irregular calcifications in comparison with the original lesion.^4^ On the MRI, Nora's lesion usually displays low signal intensity on T1-weighted sequences with uniform enhancement after gadolinium administration and a high signal intensity on T2-weighted images with its periphery having a higher signal intensity.^8^ Periosteal reaction and medullary involvement are typically absent, which is associated with the normal underlying bone and adjacent soft tissues.^8^

The cause of Nora's lesion remains unclear, but it is suggested that it may be due to reparative effects after trauma,^2^ as histologically the ossification in the lesion is irregular resembling a callus. It has also been thought of as part of the spectrum between florid periostitis and turret exostosis,^6^ with each representing a different stage of the proliferative periosteal process. There are some reports, however, supporting a neoplastic process rather than a reactive process, with several genes now identified.^8^ This postulation would be supported by high and often early recurrence rate.

Recommended treatment is complete surgical excision, with en bloc negative margins. The pseudocapsule overlying the lesion and any periosteal tissue beneath the lesion should be excised, as well as decortication of any area in the underlying host bone that appears abnormal.^4^ Depending on the size and extent of the lesion, this may result in bony instability and necessitate the need for reconstructive procedures. Intralesional excision is plagued by a high local recurrence rate but preserves stability without decortication of the affected bone. Wide resection has not been advocated because of the aggressive nature of the treatment for what is essentially a benign lesion. Despite appropriate treatment, Nora's lesion has a high recurrence rate, reported as 29% to 55% within the first 2 years following the initial treatment.^1^^,^^2^^,^^6^ Treatment of recurrent lesions, however, has been shown to have a lower recurrence rate compared with treatment of the initial lesion. Treatment of recurrent lesions carries a 12% to 22% chance of a second recurrence,^1^^,^^2^ and treatment of a second recurrence carries a 7.5% chance of a third recurrence.^2^ We postulate that this could be due to recurrent lesions being more clearly defined and having a greater ossified component, making complete excision technically easier.

## CONCLUSION

This case highlights the spectrum of Nora's lesion. Radiologically and histologically, it appears consistent with a reparative process, progressing from cartilaginous proliferation resembling a benign chondroma, then developing a minor osseous component resembling Nora's lesion, and finally a more mature central osseous component and a peripheral cartilaginous component reminiscent of an acquired osteochondroma. Treatment is complete excision of the lesion and the surrounding periosteum, and recurrences are common, with long-term follow-up essential.
